# Genomic profiling of tumor initiating prostatospheres

**DOI:** 10.1186/1471-2164-11-324

**Published:** 2010-05-25

**Authors:** Maria Ana Duhagon, Elaine M Hurt, Jose R Sotelo-Silveira, Xiaohu Zhang, William L Farrar

**Affiliations:** 1 Cancer Stem Cell Section. Laboratory of Cancer Prevention. National Cancer Institute at Frederick, 1050 Boyles Street, Frederick, MD 21702, USA; 2 Laboratorio de Interacciones Moleculares. Departamento de BiologÍa Molecular y Celular. Facultad de Ciencias. Universidad de la República, Iguá 4225, Montevideo 11400, Uruguay; 3 Departamento de Genética. Facultad de Medicina. Universidad de la República, General Flores 2125, Montevideo 11800, Uruguay; 4 Laboratory of Molecular Technology. Advanced Technology Program, SAIC-Frederick, Inc. National Cancer Institute at Frederick, 915 Toll House Avenue, Frederick, MD 21702, USA; 5 Facultad de Ciencias. Universidad de la República, Iguá 4225, Montevideo 11400, Uruguay; 6 Departamento de NeurobiologÍa Molecular y Celular. Instituto de Investigaciones Biológicas Clemente Estable, Avenida Italia 3318, Montevideo 11600, Uruguay

## Abstract

**Background:**

The cancer stem cell (CSC) hypothesis proposes that a population of tumor cells bearing stem cell properties is responsible for the origin and maintenance of tumors. Normal and cancer stem cells possess the ability to grow in vitro as self-renewing spheres, but the molecular basis of this phenotype remains largely unknown. We intended to establish a comprehensive culture system to grow prostatospheres (PSs) from both cancer cell lines and patient tumors. We then used gene expression microarrays to gain insight on the molecular pathways that sustain the PS tumor initiating cell (TIC) phenotype.

**Results:**

Traditional stem cell medium (SCM) supplemented with Knockout™SR (KO) allows the propagation of monoclonal PSs from cell lines and primary cells. PSs display gene expression and tumorigenicity hallmarks of TICs. Gene expression analysis defined a gene signature composed of 66 genes that characterize LNCaP and patient PSs. This set includes novel prostate TIC growth factors (NRP1, GDF1, JAG1), proteins implicated in cell adhesion and cytoskeletal maintenance, transcriptional regulators (MYCBP, MYBL1, ID1, ID3, FOS, ELF3, ELF4, KLF2, KLF5) and factors involved in protein biosynthesis and metabolism. Meta-analysis in Oncomine reveals that some of these genes correlate with prostate cancer status and/or progression. Reporter genes and inhibitors indicate that the Notch pathway contributes to prostatosphere growth.

**Conclusions:**

We have developed a model for the culture of PSs, and provide a genomic profile that support CSCs identity. This signature identifies novel markers and pathways that are predicted to correlate with prostate cancer evolution.

## Background

There is overwhelming evidence supporting the concept that only a specific group of cells, among the cellular heterogeneity of a tumor, possesses self-renewal and multilineage differentiation potential and is, therefore, responsible for tumor development[1]. These cells, so called "tumor initiating cells" (TICs) or "cancer stem cells" (CSCs), have been documented in most circulating and solid tumors as well as in numerous established cancer cell lines[2]. The expression of adult stem cell surface markers (e.g. CD133, CD44, ESA) as well as the expression of specific embryonic stem cell genes (e.g. OCT3/4, NANOG, SOX2) is one of the hallmarks of the TIC [2]. TICs also display increased potential for anchorage-independent growth, capacity to form spheroids in vitro, and propensity to undergo epithelial-mesenchymal transition. Nevertheless, in the current paradigm, the gold standard property of a TIC is its ability to initiate and generate a tumor in immunodeficient mice. Due to their properties, tumor initiating cells are thought to be responsible for cancer chemo-resistance and relapse, and thus they represent a significant concern for cancer prognosis and therapy[1].

The isolation of TICs is based on the expression of specific cell surface markers, the ability to pump out Hoechst dye (referred as "side population" or the cells that do not retain the dye), the high aldehyde dehydrogenase 1 activity or the ability to grow in vitro as unattached spheroids in an appropriate medium [2]. Furthermore, we and others have recently proposed that invasion ability could be also used for the enrichment of TICs [3,4]. The isolation of neural stem cells and the propagation of "neurospheres" was the first breakout in the culture of adult stem cells [5]. Neurospheres are relatively undifferentiated stem cell clones and mounting data have validated their use as a self-renewal model. Using neurosphere culture strategies, unattached clusters of cells with TIC properties, called 'prostatospheres' (PS), have been grown from both malignant and non-malignant prostate tissue [6]. The isolation and culture of TICs as PSs represents a convenient model for their study, since we currently lack universal TIC surface markers, and because it allows the propagation of TICs in their undifferentiated state. However, it remains to be determined to what extent sphere culture selectively enriches for TICs. Likewise, the molecular and cellular basis of PS growth, as well as it relevance for TIC biology, have not been analyzed in depth.

Various signaling pathways, including Wnt, Notch, Hh and PI3K/AKT, have been associated with TICs [7]. Most of these pathways have previously been found to be active in both embryonic and cancer cells. In particular, canonical Notch pathway activation has been reported in normal adult stem cells [8,9] and malignancies [10,11], and has recently been connected to self-renewal of TICs [12]. Notch signaling starts with a direct interaction between cell surface ligands and Notch membrane receptor, followed by cleavage of the Notch intracellular domain (NICD) and its translocation to the nucleus where it interacts with CSL/RBPJ transcription factor and triggers the transcription of Notch target genes [12]. In addition, Notch signaling is relevant in prostate gland development, neoplasia and possible for prostate TICs [11].

Here we show that stem cell medium (SCM) supplemented with Knockout™SR serum replacement (KO) promotes the growth and serial passage of clonally derived PSs. This culture method is suitable not only for established cell lines but also for prostate cancer stem cell primary cultures (PCSC-1, PCSC-2 and PCSC-3, referred to as group as "PCSCs"), providing a valuable model for the integrated study of patient and cell line material. The TIC nature of PSs is supported by their increased tumorigenicity in mouse xenograft studies, as well as by their increased expression of several established stem cell markers.

In the search for the molecular basis of PS growth we performed genome expression studies using microarrays. We found that the promoters of the genes modulated in PSs are enriched in binding sites for known TIC transcription factors, such as c-Myc and TCF3, thereby reinforcing their TIC nature. We also identified 66 PS regulated genes that are common to LNCaP and PCSCs, and highlighted interesting molecules that have recognized roles in developmental processes, tissue remodeling, cell invasion and stem cell niche. Meta-analysis in Oncomine prostate datasets indicates that 32 of the 66 genes in this signature correlate with prostate cancer status. These genes could, therefore, represent prostate CSC markers that have still uninvestigated roles in TIC biology.

Finally, the overexpression of Jag1 in the PS signature, lead us to study the contribution of the Notch pathway to PS biology. We uncovered an overexpression of several Notch signaling genes, an increase in CSL/RBPJ activity in PSs, as well as a reduction of sphere formation as a result of gamma secretase inhibition. This evidence supports a role for the Notch pathway in PS maintenance. In conclusion, our findings support the use of PS cultures in SCM-1%KO as a model for TIC propagation and isolation that unifies cell lines and primary culture systems, and exposes new molecular actors that might be relevant for TIC biology and prostate cancer progression.

## Results

### SCM-1%KO supports monoclonal PS growth from CD44+CD24-TICs and the total cell line

**Figure 1:**
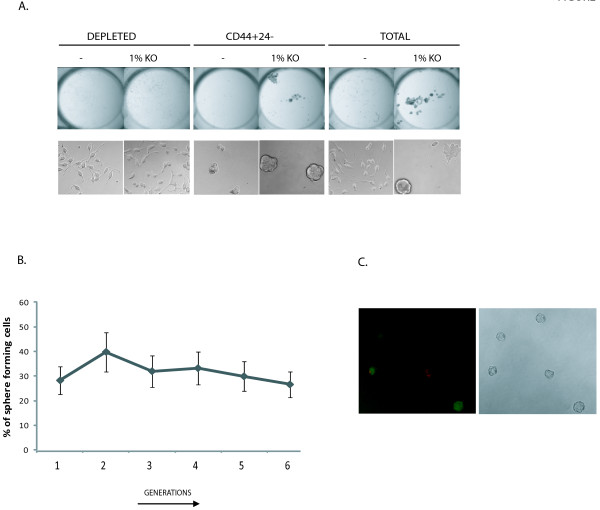
**Prostatosphere formation in LNCaP cell line**. A. Morphology of LNCaP subpopulations plated in SCM only (-) or in SCM-1%KO (1%KO). Top: 2400 dpi scans. Bottom: 20 × lens phase contrast images. B. Percentage (%) of sphere forming cells during 6 serial passages or generations (G 1-6). C. Fluorescent microscopy images of Cell-tracing experiments with 20 × objective.

Critical to the study of the TIC populations within tumors is the establishment of cultureconditions that allow putative TICs to exhibit symmetric (self-renewal) and asymmetric (differentiation) cell divisions. In order to find a comprehensive medium for the growth and maintenance of prostate TICs, we tested the ability of various media and serum replacements developed for embryonic and mesenchymal stem cell research, to support PS growth (data not shown). We found that SCM supplemented with 1% KO, promoted the formation of spheres in LNCaP as well as other prostate cell lines (data not show). In order to determine whether the PS initiating cells represent the previously characterized TICs isolated by surface markers [13], LNCaP CD44+24-stem cell population was sorted and cultured in sphere forming conditions. As shown in Figure 1A, depleted LNCaP cells (previously shown to represent the differentiated non-TIC subpopulation) attach to the flask as a monolayer whereas CD44+24-cells grow as spheres in SCM. Addition of 1% KO supplement causes an increase in the proliferation rate of the CD44+24-spheres, as indicated by the augmented diameter of the spheres.

When the bulk LNCaP cell line was cultured in SCM-1%KO medium, we observed the growth of spheres morphologically similar to those originating from the CD44+CD24-subpopulation (Figure 1A, "total"). After day 7, the cultures displayed a mixed population of adherent cells similar to those grown in RPMI-1640-10% FBS, and PSs (approximately 30%) (Figure 1B). Indeed, PSs could be serially passaged without a decrease in the percentage of sphere forming cells, which denotes the preservation of their self-renewal potential. qRT-PCR analysis of some stem cell genes (CD44, Oct3/4 and Nanog) indicated maintenance of their expression along at least eight generations (data not shown). Therefore, this culture condition seems to allow for the enrichment in TIC spheres from the total LNCaP cell line.

In order to distinguish between cell aggregation and clonal origin of these PSs, we carried out cell tracing experiments. Single cell suspensions were stained with two different dyes in separate reactions and then mixed in equivalent amounts and cultured in SCM-1%KO for 7 days. Following cell up-take, these two dyes become covalently linked to thiol groups in the cytosol and segregate with the cytoplasm. At day 7, the vast majority of the spheres have only one type of fluorescence, indicating that they have a single cell origin (Figure 1C). Two-colored spheres probably arose from peripheral adhesion of small strongly-stained dead cells. This experiment demonstrates that our culture conditions promote the clonal growth of prostatospheres.

### SCM-1%KO supports monoclonal PS growth from CD133+TICs isolated from primary cultures

Subsequently, we performed a similar study with three primary prostate cancer stem cells isolated from recurrent prostate tumors of 55-, 56- and 70-year old patients that underwent chemotherapy and radiation therapy prior to tumor extraction. Thereafter, the tumor cells were disaggregated and TIC were sorted for expression of the adult stem cell marker PROMININ1 (CD133) (personal communication, Jay Sharma). All three PCSCs grow as epithelial monolayers in the commercial media, express the TIC markers CD44, CD133, NANOG, OCT3/4, BMI1 and exhibit ALDH activity (Additional file 1A) and anchorage independent growth (Additional file 1B). Additionally, they are highly tumorigenic in NOD/SCID mice, with 100 cells producing tumors in all animals in less than eight weeks (Additional file 1C). These properties are consistent with PCSCs containing a TIC subpopulation.

**Figure 2:**
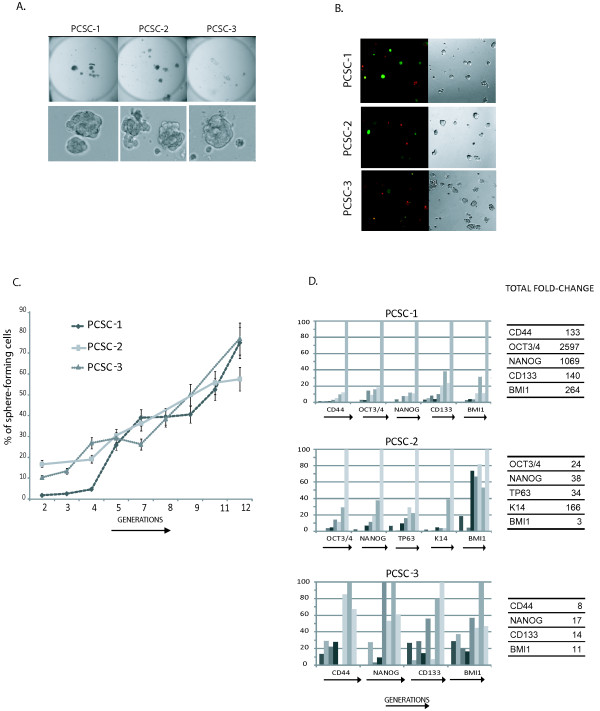
**Prostatosphere formation in PCSCs cell lines**. A. Morphology of PCSC subpopulations plated in SCM only (-) or in SCM-1%KO (1%KO). Top: 96 well plate well 2400 dpi scans, Bottom: 20 × lens phase contrast images. B. Fluorescent microscopy images of Cell-tracing experiments. 20 × lens magnification. C. Percentage (%) of sphere forming cells during 12 serial passages or generations (G 2-12). D. qRT-PCR of stem cell genes along generations. OCT3/4, NANOG, BMI1, CD44, CD133, TP63, K14 mRNA levels were assessed and only those that displayed variation along the generations are shown. In order to integrate the data into a single plot, the expression is presented as percentage of the maximum observed fold change. Rightmost tables indicate the absolute fold-change per gene and cell line.

The three primary cell cultures also form spheres when cultured in SCM-1%KO (Figure 2A). Although the proportion of stem cell markers and the anchorage independent growth (Additional file 1) correlate with tumorigenicity, the similarity in sphere forming capacity suggests that it correlates to a lesser extent. Cell tracing experiments demonstrated that PCSC's PSs are also monoclonal (Figure 2B). All three PCSC were passaged weekly for more that 12 generations (Figure 2C). As shown in Figure 2D PCSCs gained sphere-forming capacity along the generations. Furthermore, several stem cell markers were shown to be progressively up-regulated along consecutive passages. This data indicate that SCM-1%KO medium not only sustains the growth of primary PCSCs but also positively selects for TICs during passages.

### PSs are more tumorigenic than the parental cell lines

**Figure 3:**
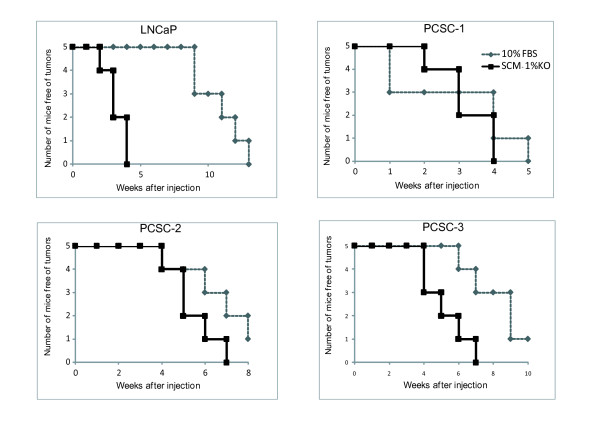
**Mouse xenograft experiments for determination of tumorigenicity**. Male NOD/SCID mice were subcutaneously injected with 100 cells from either parental (10% FBS) or sphere cell (SCM-1%KO). Mice were weekly monitored for tumor formation. Kapplan-Meier curves for LNCaP and the three PCSC cell lines are presented.

In order to determine if PSs grown in SCM-1%KO have increased tumorigenic ability, as it would be expected for TICs, we compared the tumorigenicity of the PSs and the parental cultures. LNCaP PSs grown in SCM-1%KO for one week generate palpable tumors in NOD/SCID mice in approximately four weeks in 5/5 animals (Figure 3). That is seven times faster than what was reported by our group before [13]. Likewise, purified PCSC PSs grown for one week in SCM-1%KO have an increased ability to form tumors than their parental counterparts (Figure 3). This difference in tumorigenicity seems to be indirectly proportional to tumorigenicity and stem cell marker expression of the parental cell lines. For instance, PCSC-3 parental culture is the least aggressive in mice (Additional file 1C) and the one that has the lowest expression of TIC markers (Additional file 1A). Simultaneously, it shows the largest difference in tumorigenicity between PSs and parental cell culture (Figure 3) as well as the fastest increase in stem cell gene expression (Figure 2D), suggesting that it undergoes a strong selection for TICs in this culture condition. Likewise, the PCSC-1 phenotype indicates the highest TIC content (Additional file 1), but no difference in tumorigenicity between adherent cultures and the PSs (Figure 3). In that case, a reduction in the number of injected cells would likely be necessary to detect enrichment in TIC in the PSs.

In summary, the mice xenograft experiments demonstrate that PS culture enriches in TICs both in LNCaP as well as in PCSCs.

### Gene expression analysis of prostatospheres and differentiated cells

The discovery of the molecular pathways that are required for the maintenance of self-renewing TICs may ultimately lead to targeted therapies against this unique population of tumor cells. In order to identify the molecular basis of PS biology, we carried out whole genome gene expression analysis using microarrays. Microarray data were deposited in GEO (LNCaP expression data in GSE19704 and PCSCs expression data in GSE19713). Gene expression profiles of prostatospheres growing in SCM-1%KO for 7 days were compared to the parental cell line growing in the commercial medium containing 10% FBS.

**Figure 4:**
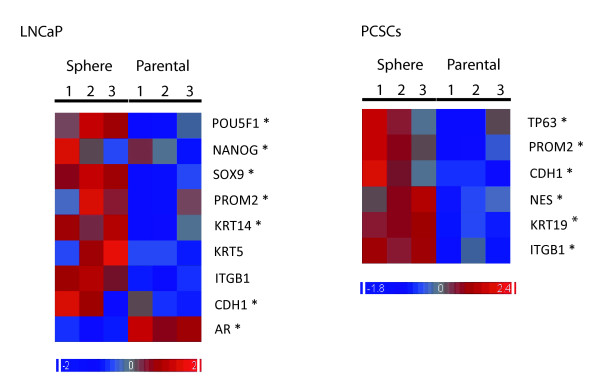
**Expression of CSC markers in PSs**. Heatmaps of the mRNA levels of literature-defined CSC markers in LNCaP and PCSCs. Each heatmap compares spheres growing in SCM-1%KO (spheres) and adherent cultures growing in commercial medium supplemented with 10% FBS (parental). Left panel shows three biological replicates of LNCaP cell line. Right panel shows the three individual primary cell cultures from PCSC-1 (1), PCSC-2 (2) and PCSC-3 (3) as indicated, and the result is the average of the two replicates for each cell line. Only genes that show a significant change (p > 0.05) are included in the heatmap.

Several genes that were previously reported to be related to TIC maintenance were found to be up-regulated in SCM-1%KO cultured PSs (Figure 4A). Microarray analysis shows that CDH1, PROM2 and ITGB1 are upregulated in both LNCaP and PCSCs. Although not detected by microarrays, further qRT-PCR assessment also showed that NES and NANOG are significantly upregulated in both (Additional file 2). AR is downregulated in the PSs derived from the androgen-dependant/sensitive LNCaP cell line. For PCSCs, the AR protein was undetectable by western blots although expression of AR mRNA was measurable by qRT-PCR (data not shown). Due to previously published reports on PROM2 and SOX9 expression in prostate progenitor cell [14,15] we also addressed their gene expression levels (Figure 4). The changes in stem cell markers identified by microarray were further confirmed by qRT-PCR (Additional file 2A for LNCaP and 2B for PCSCs). Therefore, our gene expression study confirms that PSs preferentially express already described, as well as potentially novel, TIC genes.

Due to the enormous interest in the use of well established cell lines as model systems for cancer stem cell research, we sought to find the commonalities between LNCaP PSs and the primary prostate cultures by gene expression analysis. We found a total of 845 genes similarly and significantly (≥1.8-fold change and p ≤ 0.05) modulated in all three PCSCs and 1454 in LNCaP PS. Among these, only 66 were regulated in the same direction in both the PCSCs and LNCaP gene lists. In Table 1, the genes are classified according to their function and the average fold-change observed in PCSCs and LNCaP is also shown. In order to confirm the microarray data, four genes were assessed by qRT-PCR (Additional file 2C). Although changes in the abundance of individual mRNAs may explain a particular biological situation, the concerted action of a group of genes is a more robust means to understand the molecular processes and regulators that drive a given phenotype [16]. Core Analysis of the 66 gene signature in Ingenuity Pathways Analysis (IPA, http://www.ingenuity.com) yielded Cell Death and Cellular Movement as the two top categories (p ≤ E-07), followed by Cancer and Reproductive system Disease (p ≤ E-06) (Additional file 3). In addition, most of the tissue specific categories found in this analysis represent mesodermal derived tissues (connective, cardiovascular, lymphoid, gastro-intestinal) (Additional file 3).

**Table 1:** List of 66 genes modulated in PSs classified by their function.

FUNCTION		Gene Name	Symbol	PCSC	LNCaP
**Adhesion**	Cell-Cell	major histocompatibility complex, class II, DM alpha	HLA-DMA	2.0	2.2
		cadherin 3, type 1, P-cadherin (placental)	CDH3	2.1	2.0
		HLA complex group 4	HCG4	-1.9	-1.9
		activated leukocyte cell adhesion molecule	ALCAM	-2.3	-2.3
	Cell-Matrix	Kallmann syndrome 1 sequence	KAL1	-2.0	-2.2
	Extracellular matrix	collagen, type VI, alpha 1	COL6A1	1.8	2.7
	Tight junction	claudin 3	CLDN3	2.1	1.9
**Cell cycle**	Protein biosynthesis	MYC induced nuclear antigen	MINA	-1.9	-1.9
	Regulation	cyclin E2	CCNE2	-2.1	-2.7
**Cytoskeleton**	Actin binding	tropomyosin 1 (alpha)	TPM1	-2.4	-2.1
		gelsolin (amyloidosis, Finnish type)	GSN	2.1	1.9
	Regulation	Src homology 3	SGEF	-2.4	-2.1
**Growth factor**	Receptor	neuropilin 1	NRP1	8.7	2.8
	Secreted	growth differentiation factor 15	GDF15	8.0	2.2
**Metabolism**	Metals	metallothionein 1G	MT1G	-4.1	-1.9
		metallothionein 1H	MT1H	-4.4	-1.8
		metallothionein 2A	MT2A	-4.8	-1.8
	Carbohydrate	UDP-N-acteylglucosamine pyrophosphorylase 1	UAP1	-3.1	-2.1
	DNA	RAD18 homolog (S. cerevisiae)	RAD18	-1.9	-1.9
	Enzyme	methionine sulfoxide reductase B3	MSRB3	-2.1	-2.4
	Hormone	membrane metallo-endopeptidase	MME	-3.0	-2.5
	Sugar biosynthesis	lectin galactoside-binding 8 galectins	LGALS8	-2.2	-2.0
		mannosidase, alpha, class 1C, member 1	MAN1C1	1.9	1.8
	Protein biosynthesis	STAM binding protein-like 1	STAMBPL1	-2.0	-2.1
		cysteinyl-tRNA synthetase	CARS	-2.2	-1.9
		glycyl-tRNA synthetase	GARS	-2.2	-2.1
		tryptophanyl-tRNA synthetase	WARS	-2.5	-2.8
		heat shock 60 kDa protein 1 (chaperonin)	HSPD1	-2.1	-3.4
	Protein stability	YOD1 OTU deubiquinating enzyme 1 homolog	YOD1	**-2.0**	**-2.1**
**Protein binding**		tissue factor pathway inhibitor	TFPI	2.4	1.8
		neuron navigator 3	NAV3	-5.5	-2.3
**Signaling**	Hormone	relaxin 2	RLN2	-1.8	-2.0
	Hormone Receptor	prostaglandin E receptor 4 (subtype EP4)	PTGER4	-3.6	-2.9
	Integrin	sema, immunoglobulin (Ig) and short basic domain	SEMA3B	1.9	1.8
	Kinase	dual specificity phosphatase 6	DUSP6	2.0	2.2
	Ligand	jagged 1 (Alagille syndrome)	JAG1	4.0	3.2
	Membrane traffic	synaptojanin 2	SYNJ2	-2.0	-2.3
	Secreted	clusterin	CLU	2.8	2.3
		amyloid beta (A4) precursor-like protein 2	APLP2	2.7	2.0
		c-mer proto-oncogene tyrosine kinase	MERTK	2.1	2.0
		RIO kinase 3 (yeast)	RIOK3	-1.9	-2.2
**Transcription**	Activator	c-myc binding protein	MYCBP	-2.4	-2.4
	Activator	v-myb myeloblastosis viral oncogene homolog (avian)-like 1	MYBL1	-3.4	-2.0
	Inhibitor	inhibitor of DNA binding 3	ID3	-6.7	-3.1
	Inhibitor	inhibitor of DNA binding 1	ID1	-10.2	-5.2
		E74-like factor 3	ELF3	2.0	2.5
		v-fos FBJ murine osteosarcoma viral oncogene homolog	FOS	4.8	2.5
		Kruppel-like factor 2 (lung)	KLF2	2.1	1.8
		CCAAT/enhancer binding protein (C/EBP), gamma	CEBPG	-1.9	-2.0
		E74-like factor 4 (ets domain transcription factor)	ELF4	-2.3	-1.9
		zinc finger and BTB domain containing 38	ZBTB38	-2.5	-2.0
		Kruppel-like factor 5 (intestinal)	KLF5	-2.8	-2.0
**Translation**	regulation	quaking homolog, KH domain RNA binding (mouse)	QKI	-2.9	-1.9
		eukaryotic translation initiation factor 2, subunit 1 alpha	EIF2S1	-1.9	-2.0
**Transport**	Integral	Transmembrane channel-like 4	TMC4	1.8	2.2
	Zinc	solute carrier family 39 (zinc transporter), member 10	SLC39A10	2.3	2.5
		solute carrier family 25, member 30	SLC25A30	-1.8	-2.3
		solute carrier family 7, member 5	SLC7A5	-2.3	-2.3
		Hbc647 mRNA sequence	SLC30A1	-12.8	-2.3
**Unknown**		FLJ35024, C3orf59, CCDC113, C5orf35, CCDC50, CCDC76, LOC440731			

prostatospheres classified by their function.

In order to predict which transcription factors might be responsible for the maintenance of the spheroid state, we used the Molecular Signatures Database (MSigDB, http://www.broadinstitute.org/gsea/msigdb/index.jsp) to search overrepresentation of transcription factor binding sites in the promoters of the PS genes modulated ≥2-fold at p ≤ 0.01 (473 genes of PCSCs and 443 in LNCaP). Of the five common TF predictions (Additional file 4), MYC and TCF3/E2A, MYOD and YY1 are well known somatic stem cell transcription factors, whereas the function of REPIN1 is still unknown. Canonical Wnt pathway transcriptional regulators (as Tcf1, Lef1, Tcf8) and other MYC associated TFs (as MAZ) were also found to be overrepresented in the promoter regions of the specific PS datasets (Additional file 4).

### Meta-analysis for Correlations of PS genes and prostate cancer status and progression

**Figure 5:**
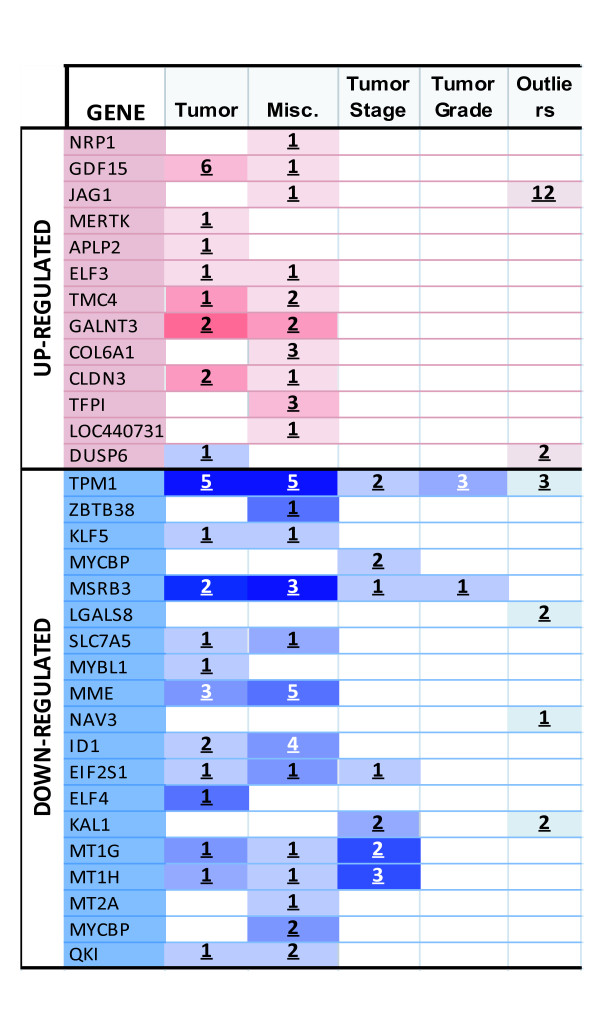
**Meta-analysis of prostate cancer microarray datasets with PS 66 gene set**. Differential activity map of the PS genes that correlate with prostate cancer studies in Oncomine microarray database. Columns categorize the different type of studies discriminated by the type of sample from which the gene expression data were collected. For instance, "tumor" refers to cancer status comparing normal vs. tumor tissue data, and cancer progression includes tumor stage or grade. Miscellaneous refers to other types of studies, including primary vs. metastatic, androgen dependence vs. independence, recurrence or drug resistance sample groups. Outlier analysis is based on Cancer Outlier Profile Analysis (COPA), which is intended to detect gene modulation in a small subset of samples that would not otherwise be detected by the Student's t-test. Red/blue colored boxes represent overexpression/underexpression of the indicated gene. Color intensity indicates the statistical significance of the correlation. The color gradient is based on the most significant profile within each cell. The number of studies in which the correlation was found is represented by the number inside each box.

Since TICs are proposed to be fundamental for tumor maintenance, invasion, metastasis and drug resistance, we investigated if the 66 PS genes were correlated to prostate cancer status or progression using the Oncomine database analysis tool [17-20]. We interrogated the 66 gene list in all prostate cancer studies available in Oncomine. Interestingly, 32 out of the 66 genes are correlated with prostate cancer in at least one prostate cancer profiling study (Figure 5). Furthermore, the vast majority of these genes associate with prostate cancer in more than one study, which reinforces their putative significance for this disease. TMP1 and GALNT3 display the most significant correlation among the list, as indicated by the intensity of the colors of the corresponding boxes. Only the downregulated genes correlate with tumor stage and grade. The finding of these numerous significant correlations with the clinical set underscores the importance of our PS signature for prostate cancer status and progression.

### Notch pathway is upregulated in PSs and may contribute to PS maintenance

**Figure 6:**
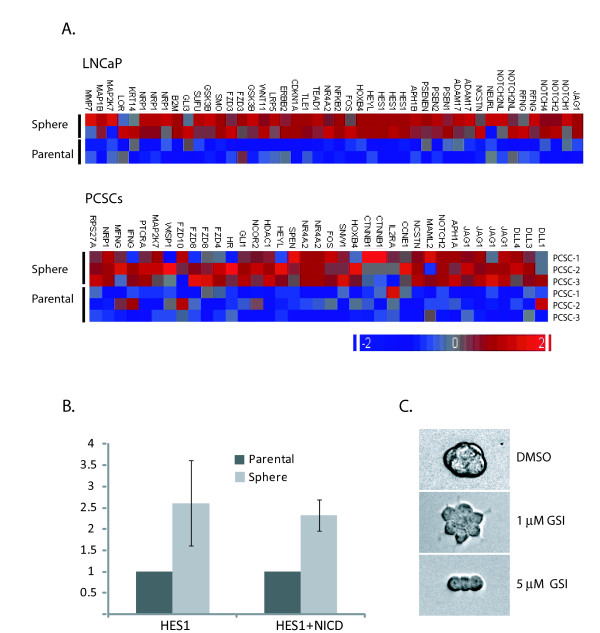
**Notch pathway activity in PSs**. A. Heatmap of selected significantly modulated (p ≤ 0.05) Notch pathway genes in LNCaP and PCSCs. Top panel shows two biological replicates of LNCaP cell line. Right panel shows the average of the two replicates for each primary cell cultures from PCSC-1 (1), PCSC-2 (2) and PCSC-3 (3), as indicated. B. Reporter gene analysis on LNCaP cells transfected with pGL4.17-HES1 (HES1) and pBOS-NCID (NCID). Error bars represent the standard deviation of three independent experiments. Y axis shows the relative normalized reporter activity ratio between PS/parental cell line. C. LNCaP cells cultured for 7 days in SCM-1%KO in the absence (DMSO) or the presence of the indicated concentration of the γ-secretase inhibitor (GSI). Representative pictures of each the culture. 20 × lens phase contrast.

The significant increase in JAG1 expression observed in our microarray analysis, and subsequently confirmed by qRT-PCR (Additional file 2C), together with the well documented role of the Notch pathway in the maintenance of "stemness", lead us to study the involvement of this pathway in SCM-1%KO PS growth and maintenance [12]. Initially, we queried the microarrays for genes that belong to the canonical Notch pathway and genes that participate in its crosstalk with the Wnt and Hh pathways[21]. As shown in Figure 6A, many of these genes are up-regulated in LNCaP and PCSCs PSs. Additionally, reporter gene analysis using the promoter of HES1, whose transcription is driven by CSL, evidenced a 2-3-fold increase in reporter activity in LNCaP PSs relative to the parental cell line (Figure 6B). Co-expression of NICD stimulates HES1 promoter activity in a similar magnitude thus supporting the specificity and sensitivity of the reporter assay. Finally, we found that the γ-secretase inhibitor (L-685,458, Calbiochem) not only inhibited the growth of LNCaP PSs but also induced an adherent morphological phenotype (Figure 6C). This data indicate a higher activity of the Notch pathway in PSs, and a role of this signaling cascade in PS growth.

## Discussion

The notion that progenitors as well as TICs can grow in vitro as spheroids has been extensively documented for several tissues types. However, the lack of consensus culturing conditions has hampered the elucidation of the molecular pathways that sustain this phenotype. In an attempt to find a comprehensive medium suitable for the selection and propagation of prostate TICs, we tested various embryonic stem cell media. We found that SCM supplemented with Knockout™SR (Gibco-Invitrogen) was able to select TICs, sustaining the growth of PSs not only from normal and cancer cell lines, but also from early passage prostate tumor cell cultures. We demonstrated that these PSs have a monoclonal origin, a relevant aspect that is seldom addressed in the cancer stem cell field. The stem cell identity of these prostatospheres was supported by their extensive in vitro self-renewal potential in the "neurosphere assay", the expression of known prostate TIC markers and the increased tumorigenicity compared to the parental cell line. Indeed, SCM-1%KO culture seems to select in favor of TICs along passages, as was previously observed for normal mouse prostate progenitor cells [22]. It is therefore possible that this culture system provides a means for the long-term enrichment of TICs.

Our analysis of stem cell markers evidenced that PSs preferentially express some classic embryonic stem cell (OCT3/4, Nanog) and TIC (TP63, CDH1, ITGB1, KRT14, KRT5) markers. The down-regulation of AR mRNA in LNCaP is in agreement with the reported reduction in AR expression in prostate TICs[22]. Our data also suggest that prominin-2 could be an interesting new marker for prostate cancer TICs (Figure 2A). PROM2 is a paralogue of PROM1 that is expressed in the prostate and co-localizes with Promin1 in epithelial cells [23]. Indeed, PROM2 protein is enriched in cells located in the basal compartment of the glandular epithelium where only few cells were found to be positive for PROM1 [14]. Another attractive stem cell marker candidate is the HMG-box class transcription factor SOX9, which is expressed in embryonic and somatic progenitor cells [24]. Moreover, Sox9 is required for prostate development [15] and its expression was found to be restricted to basal epithelium in the normal human adult [25]. Furthermore, prostate tumors express this TF and its expression increases in relapsed hormone refractory tumors[25]. The same report showed that Sox9 is regulated by β-catenin and affects prostate cancer cell proliferation, angiogenesis and invasion; in particular in the LNCaP cell line. Finally, our data also support the observation that NANOG is a key regulator of prostate TICs [26].

In order to interpret our microarray data, we carried out several analyses. IPA highlighted cell death and cellular movement as the principal gene categories regulated in PSs, which is consistent with the modulation of self-renewal and with the morphological differences between monolayer and spheroid growth. The finding of an enrichment in embryonic TFs related to MYC and TCF-3, which are indeed regulators of cancer stem cell pathways [27,28], reinforces the TIC identity of the PSs. In fact, our group and others recently provided evidence in favor of a role for Wnt signalling in prostate TICs [13,29,30]. MYOD1 has been recently implicated in myoblast maintenance, and, in combination with TCF3, it inhibits myoblast differentiation leading to Rhabdomyosarcomas [31]. YY1 is a pleiotropic transcription factor expressed in prostate glandular epithelium and basal cells, and its expression is positively correlated with prostate cancer metastasis (reviewed in [32]). Indeed, it is well documented to be involved in specification of mesodermal lineages [32]. The prediction of mesenchymal transcription factor activity (MYOD1 and YY1) could be indicative of an epithelial-mesenchymal transition, which has lately been related to tumor progenitor cell plasticity [4,33]. In summary, the four known TF predicted to be modulated in PCSCs and LNCaP PSs are known to be active in adult stem cells.

We also defined a set of 66 genes that are analogously and significantly regulated in LNCaP and PCSCs (Table 1). It includes several cell adhesion and cytoskeletal proteins, signaling molecules, transcriptional regulators and metabolic factors. The role of most of these factors in TIC biology has not yet been studied. To date, three complete gene expression datasets of putative prostate TICs have been published. Dubrovska et. al. reported the genomic profiling of PSs derived from DU145 and PC3 cell lines [34]. Strikingly, 11 genes of our 66 gene signature (TFPI, NAV3, MYCBP, MSRB3, MERTK, LOC440731, ID3, ID1, GSN, CCDC50 and C3orf59) overlap with their dataset. Analysis of CD133+ vs. Alpha2lowprostate tumor cell array data published by Birnie et al. reveal a set of four genes common to our signature (JAG1, APLP2, SEMA1A and RIOK3)[35]. Comparison of the gene expression profiles of CD133+ and CD133- cells isolated from hormone-refractory prostate cancer biopsies, published by Shepherd et al. [36], shows that EIF2S1, RAD18, QKI, MAN1C1 and MERTK are significantly regulated in the direction observed in our signature. The small overlap between the studies might be explained by the differences in the methods used for the isolation and culturing of TICs.

Remarkably, 31 of the 66 genes correlated to prostate cancer in our study of Oncomine dataset. Among them, several have already been reported to be associated with prostate cancer. Neuropilin1, for instance, has been proposed as a marker of aggressiveness of prostate carcinoma, as well as other tumor types [37]. Its ability to modulate tumor cell invasion and migration represents one interesting commonality with cancer stem cells [38]. Likewise, GDF15 has been associated with the progression of diseaseto metastasis [39] and has also been proposed as a prostate cancer biomarker [40]. Its capacity to inhibit proliferation is consistent with the quiescence of TICs. In addition, JAG1, one of the ligands for the Notch receptor, is proposed as a prostate cancer marker [41]. Five other PS up-regulated genes (MERTK^13^, APLP2 [42], CLDN 3 [43], DUSP6 [44] and TFPI [45]) have been found to be overexpressed in prostate cancer and many other tumor types, but their role in tumor biology is less understood. Finally, two genes are associated with non-prostatic neoplasms: ELF3 with breast cancer [46] and COL6A1 with astrocytoma [47]. In addition, several of the 18 down-regulated genes have been previously shown to be decreased in prostate cancer. Kruppel-like factor 5, for example, is considered a potential tumor suppressor in prostate and breast cancer [48] as well as tropomiosin1 in several tumors types [49]. Of note, the tumor suppressor candidate ELF4 facilitates entrance to the cell cycle of quiescent hematopoietic stem cells [50]. Similarly, expression of the cell adhesion protein KAL1 was found to be inversely correlated with metastatic capacity in prostate and other tumor types [51]. Likewise, several studies demonstrated a significant negative correlation between expression of metallothioneins and cancer/progression in prostate tumors and cell lines [27]. Finally, MME is contained in a potential prostate cancer susceptibility locus in prostate cancer ACI rat models [52]. Galectins [53], NAV3 [54] and QKI [55] are also correlated to cancer types other than prostate. Lastly, many genes of this 66 gene list have not been extensively studied yet.

Contrary to the oncogenic role assigned by the literature [56,57], our analysis in Oncomine indicates that SLC7A5 and ID1 are negatively correlated with cancer progression. The explanation for this discrepancy is unclear. In support of our findings, ID1 and ID3 downregulation has been also observed in another TIC profiling study [34].

Due to the over-expression of the Jag1 Notch1 receptor in PSs, we addressed the involvement of Notch signaling in PS growth. Gene expression analysis of Notch signaling cascade, reporter gene studies, and pathway inhibition experiments presented here indicate a higher activity of the Notch pathway in PSs as compared to the parental cell lines. In support of our hypothesis, Bisson et al. recently reported that the LNCaP derivative, C4-2B, which displays about five times higher Notch activity than original LNCaP cells [58], expresses more stem cell markers and has higher sphere forming ability than LNCaP [29]. It is also worth mentioning that some of the genes overexpressed in PSs (including NRP1 [59], SOX9 [60], ID1[61] and YY1 [62]) are thought to be regulated by Notch signaling. Altogether this data support the hypothesis that the Notch pathway is contributing to the PS phenotype.

## Conclusion

We presented the isolation and propagation of monoclonal cancer PSs from several cell lines and early passaged tumor cells. In agreement with the TIC identity of these PSs, we demonstrated that they bear the fundamental TIC traits of marker expression, self-renewal and tumorigenicity. The culture medium used in our study could provide a new means for the standardization of prostate TIC cultures, and may possibly reduce the difficulties of the comparison between laboratory cell lines and patient material.

Furthermore, we propose new candidate prostate TIC markers (PROM2 and SOX9) as well as potential biomarkers for prostate cancer status and progression that may be related to the TIC phenotype. Further studies will be needed to understand the role of each one of them. Finally, we show evidence in favor of a role of the Notch pathway in the maintenance of PS phenotype.

## Methods

### Cell culture

LNCaP lymph node metastatic human prostate carcinoma cell line was purchased from ATCC (ATCC^® ^Number CDL-1740™) and cultured following provider's instructions. LNCaP cells used in our study are late passages and have been cultured without addition of androgens.

PCSC-1, PCSC-2 and PCSC-3 prostate cancer stem cells (PCSCs) were purchased from Celprogen^®^. They were isolated as primary cells from human adult prostate cancer tissue based on the expression of the adult stem cell marker Prominin1 (CD133) (personal communication, Jay Sharma). The cells were cultured with the medium, extracellular matrix coated flasks and guidelines provided by the company.

### Prostatosphere culture and assay

In order to obtain prostatospheres from either LNCaP or PCSCs, exponentially growing cultures were dissociated to single cells by standard trypsinization, washed three times with PBS and plated in SCM medium (DMEM:F12 plus 10 ng/mL bFGF, 20 ng/mL EGF, 5 mg/mL insulin, and 0.4% BSA) supplemented with 1% KO serum replacement (Invitrogen/Gibco, p/n 10828-028) at a density of 2500 cells/mL in tissue culture treated flasks. After approximately 7 days, the unattached spheres were removed from the flasks, washed in PBS, incubated in trypsin and gently pipetted until single cells were obtained, as determined by microscopic observation. Trypsin action was immediately inhibited by the addition of Trypsin neutralizing solution (Lonza Ltd., p/n CC-5002). Following centrifugation, the cells were resuspended at the same density in SCM-1%KO medium.

Seven day old spheres were counted and analyzed using a GelCount™ automatic plate scanner (Oxford Optronics) and GelCount Version 0.025.1 software (Oxford Optronics). Plates were scanned at 2400 dpi and the colony detection algorithm was customized for each cell type and culture time.

### Flow cytometry of LNCaP

Flow cytometric analysis of LNCaP cells was performed as previously described [13] using antibodies against CD44 (Caltag human CD44 antibody -PE) and CD24 (Caltag human CD24 antibody -FITC) at 10 μl antibody per one million cells. Forward and side scatters were used to select live cells and scatter and pulse width to select single cells. Control scatters of unstained cells and isotype specific stains were used to set the gates. Since the number of CD44+24-cells obtained in each sorting is extremely low, no assessment of sorting efficiency was performed.

### Gene expression analysis

Total RNA isolation was performed using miRNAeasy (Qiagen). cDNA was synthesized with SuperScript III First-Strand Synthesis System for RT-PCR (Invitrogen, p/n 18080-05) using random hexamers and following manufacturer instructions.

Analysis of gene expression by qRT-PCR were performed using TaqMan gene expression assays (Applied Biosystem) in a StepOne Real time PCR machine (Applied Biosystem) using the comparative Ct procedure. For the analysis of prostate CSC markers along the generations, the RNA isolations were carried out with Cell-to-Ct kit (Applied Biosystem) and the resulting cDNA was pre-amplifed prior to the qRT-PCR using the TaqMan PreAmp Master Mix Kit (Applied Biosystem, p/n 4384267) and a pool of stem cell TaqMan Gene Expression Assays for 10 cycles. 18S RNA was not amplified, and was used as the RNA content reference control.

For microarray analysis, RNA integrity was assessed with the Agilent RNA 6000 Nano LabChip Kit (Agilent, p/n 5067-1511) and only samples with RNA integrity numbers (RIN) > 9 were used. Ten ug of total RNA from LNCaP cells were labeled by reverse transcription with Superscript II (Invitrogen) and oligo-dT in the presence of Cy3-dUTP for Universal RNA reference control (Universal Human Reference RNA Stratagene p/n 740000) or Cy5-dUTP for the samples. Whole Human Genome Oligo Microarray 4 × 44 K format gene expression arrays (Agilent, p/n G4112F) were employed for gene expression studies. The hybridization and washes were performed using Agilent reagent and protocols (G2534-90001) and the slides were scanned on a GenePix 4000B scanner (Molecular Devices, Sunnyvale, CA, USA).

PCSC gene expression wasassessed by Affimetrix U133 arrays (Human Genome U133 Plus 2.0 Array; Affymetrix, p/n 900467). In this case, 1 ug of RNA was reverse transcribed with T7-oligo(dT) primer and labeled with biotin using Affymetrix 3' One Cycle Target Labeling kit, following manufacturer's instructions. Three replicates of each group were prepared, labeled, and hybridized to Affymetrix human U133 plus 2.0 GeneChip and scanned on Affymetrix GeneChip scanner 3000. Data were collected using Affymetrix GCOS software.

Statistical and clustering analyses were performed with Partek Genomics Suite software using RMA normalization algorithm for Affymetrix arrays and Loess for Agilent arrays (Partek, Inc.). Differentially expressed genes were identified with ANOVA analysis of the replicates. Unless specifically indicated, genes that are up-or down-regulated more than 2-fold and with a p < 0.01 were considered. Differentially expressed genes were identified with ANOVA analysis of the replicates. For statistical analysis, the three PCSCs were considered as replicates. Due to the use of different array platforms, the gene lists were independently generated for LNCaP and PCSCs. Pathways and signature analysis were also performed in an independent way for each platform. Commonalities in Gene Expression changes, modulated pathways and gene signature matches were obtained as the intersection of the two independent lists (LNCaP and PCSCs).

### Cell tracing analysis

To assess the clonal origin of each prostatosphere, dissociated single cells were stained with the fluorescent dye CellTracker green or CellTracker red (Molecular Probes/Invitrogen) at a concentration of 5 μM in separate reactions, following manufacturer instructions. Equal amounts of the two reactions were immediately mixed and plated at a density of 2500 viable cells/mL in SCM-1%KO. Pictures were taken at day 6 using an Olympus IX70 inverted microscope equipped with a triple 41001 FITC EGFP/BODIPY filter (Ex: HQ480/40, Em: HQ535, BS: Q505LP).

### Mice xenograft for tumorigenicity determinations

NCI-Frederick is accredited by AAALAC International and follows the Public Health Service Policy for the Care and Use of Laboratory Animals. Animal Care was provided in accordance with the procedures outlined in the 'Guide for Care and Use of Laboratory Animals' (Institute of Laboratory Animal Resources, 1996). Cell cultures were trypsinized and resuspended in SCM-1%KO at the indicated cell density. Fifty μl of the suspensions were combined with 50 μl of BD Matrigel™ Matrix Basement Membrane (BD Bioscience, p/n 354234) on ice and subcutaneously injected into eight-week old male NOD/SCID mice (Jackson Labs). Tumor growth was monitored weekly and dimensions were measured by calipers. Animals were euthanized and tumors were dissected when they reached 1.5 sq.mm.

### Soft agar assay

Soft agar assays were performed in 96 well plates using the CytoSelectTM 96-well Cell Transformation Assay (Soft Agar Colony Formation) kit (Cell Biolabs, Inc., p/n CBA-130) following manufacturer instructions. Five hundred to five thousand cells per well were used and the plates were incubated for 10 days at 37°C, 5% CO_2_. Cells were stained with Neutral Red (Sigma) for 1 hour, scanned in a GelCount plate scanner (Oxford Optronics) at 2400 dpi and analysed using GelCount software.

### Meta-analysis for Correlations of PS genes and prostate cancer status and progression

For the assessment of correlation between PS gene set and prostate cancer status and progression we used Oncomine database analysis tool http://www.oncomine.org developed by Chinnaiyan and colleagues [63]. The P-Value Threshold to generate the map was set at 1E-4, whereas the Outlier Rank Threshold level used to generate the Outlier column was 50. Student's *t*-test was used for analyzing differences between publisheddatasets in the database.

### Transfection and reporter gene assay

For LNCaP reporter gene experiments, cultures growing in exponential phase were trypsinized and single cell suspensions were plated in a standard tissue culture 24 well-plates at a density of 40000 cells/well, and then cultured overnight in a 37°C incubator with 5% CO_2_. The pGL4.17-HES1 plasmid bears the -194 to +160 promoter fragment of the human HES1 gene in the MCS of pGL4.17[*luc2*/Neo] vector (Promega, p/n E6721). The fragment was excised from a pGL2 basic-HES1 plasmid [64] generously provided by Ndiaye Delphine (Pasteur Institute, France). pBOS-NICD, a plasmid encoding the Notch intracellular domain (NICD), was a gift from Gerry Weinmaster (UCLA, USA). pGL4-75 (hR*luc*/CMV) (Promega, p/n E6931) expresses Renilla luciferase gene under the control of the CMV promoter. Plasmids were transiently transfected using Fugene 6 (Roche, p/n 11 815 091 001) following manufacturer's instructions. Two hundred ng of pGL4- and 50 ng of pBOS-NICD and control pGL4-76 were used in each reaction. All assays were performed in triplicate. After 24 h of incubation the cells were lysed and the lysate was transferred to a 96 well plate. Reporter protein activity was measured in an Infinite 2000^® ^Series plate reader (Tecan) using the Dual-Glo^® ^Luciferase Assay (Promega, p/n E2920) following manufacturer's protocol.

## Abbreviations

CSC: Cancer Stem Cell; IPA: Ingenuity Pathway Analysis; KO: KnockOut serum replacement; NICD: Notch intracellular domain; PCSC: Prostate Cancer Stem Cell; PS: Prostatosphere; SCM: Stem Cell Medium; TIC: Tumor Initiating Cell; TF: Transcription Factor.

## Authors' contributions

MADS conceived and designed the study and carried out the collection and/or assembly of data, data analysis and interpretation, manuscript writing and editing. EMH participated in the design, provision of study material and manuscript improvement and also provided administrative support. JRS participated in microarray experiments, design and analysis. XZ was involved in data collection and offered administrative support. WLF participated in the conception, design and final approval of manuscript. All authors read and approved the manuscript.

## Supplementary Material

Additional file 1 **Characterization of PCSCs tumor cell lines**. A. Expression of stem cell makers at the protein level. OCT3/4, NANOG, BMI1, SSE4, AR and KRT14 were assessed by western blot using total extracts and infrared conjugated antibodies in a LICOR's Odyssey Infrared Imaging System. Positive and negative signals are indicated by "+" and "-"respectively. Percentage of cells expressing CD44 and CD133 surface markers was determined by flow cytometry and is expressed as percentages of the total cell line. Aldehyde dehydrogenase (ALDH) activity was assessed with ALDEFLUOR^® ^kit (StemCell Technologies Inc.) and is expressed as percentage of positive cells.
B. Anchorage independent growth of PCSCs. Soft agar assays were carried out in 96 well plates using Cell Biolabs CytoSelect™ 96-well Cell Transformation Assay. 500, 2500 and 10000 cells were plated in triplicates. Upper panel shows 2 × lens low magnification pictures of the entire well (of a 96 well plate) at 2500 cells density after 10 days in culture. Lower plot presents the averaged relative fluorescent units for the three cell densities assessed at 10 days in culture.
C. Kapplan-Meier survival curves of NOD/SCID mice xenograft experiments injecting parental PCSCs. 100, 1000, 10000 and 300000 cells of PCSC-1, PCSC-2 or PCSC-3 were used per injection as described in Materials and Methods. Click here for file

Additional file 2 **qRT-PCR validation of selected genes**. Stem cell markers in LNCaP (A) and PCSCs (B) and the 66 gene signature genes (C). The data for each gene is the average of at least two biological replicates. Error bars represent averaged standard error in the measurements of a given gene. Click here for file

Additional file 3 **Most significant (p ≤ E-4) functional categories represented in the 66 genes PS gene set**. The numbers indicate the number of genes in each category. Click here for file

Additional file 4 **List of TF binding sites enrichments retrieved from MSigDB**. Upper chart presents the five PSs that are common to LNCaP and PCSCs (≥2-fold change and p ≤ 0.01). Lower chart describes those TF that are specific for each type of cell line. Click here for file
