# Supplementary material for: Genomic profiling of tumor initiating prostatospheres
Source: BMC Genomics. 2010 May 25;11:324. doi: 10.1186/1471-2164-11-324 (PMC2900264; doi:10.1186/1471-2164-11-324)

A. Expression of Stem Cell Markers

	PCSC-1	PCSC-2	PCSC-3
OCT3/4	+	+	+
NANOG	+	+	+
BMI1	+	+	+
K14	+	+	+
SSE4	+	+	+
AR	-	-	-
CD44	50%	40%	>30%
CD133	10%	1%	1%
ALDH	8%	11%	3%

B. Anchorage independent growth

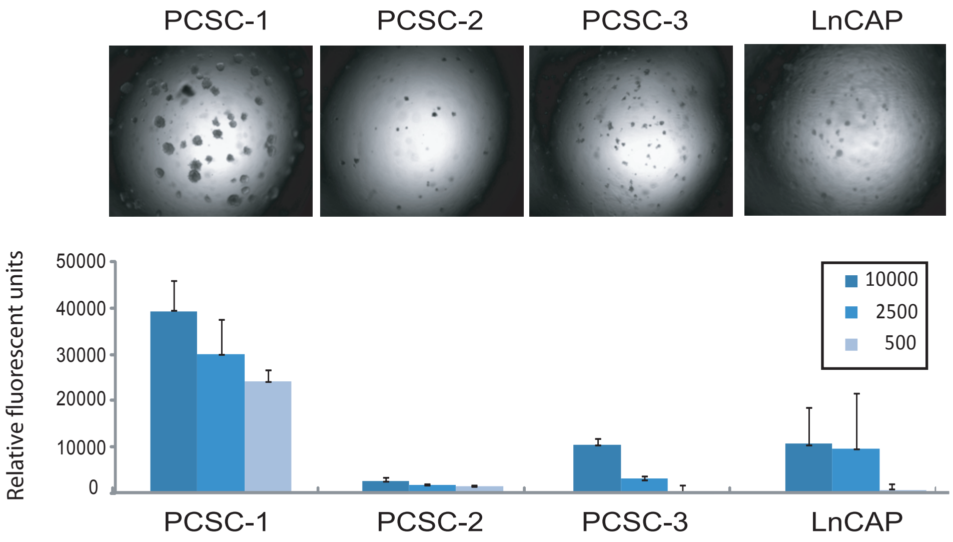

C. Tumorgenicity

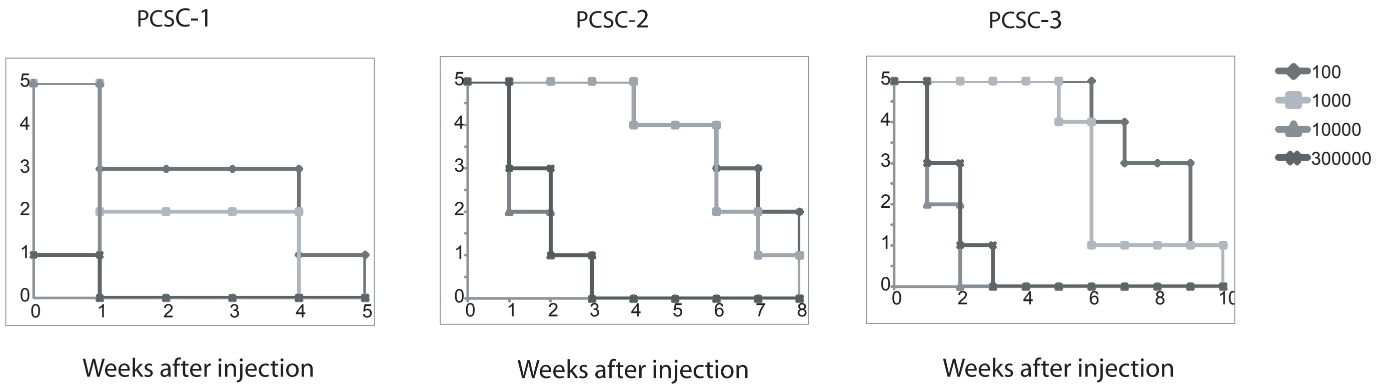

Supplement: Additional file 1 — Characterization of PCSCs tumor cell lines. A. Expression of stem cell makers at the protein level. OCT3/4, NANOG, BMI1, SSE4, AR and KRT14 were assessed by western blot using total extracts and infrared conjugated antibodies in a LICOR's Odyssey Infrared Imaging System. Positive and negative signals are indicated by "+" and "-"respectively. Percentage of cells expressing CD44 and CD133 surface markers was determined by flow cytometry and is expressed as percentages of the total cell line. Aldehyde dehydrogenase (ALDH) activity was assessed with ALDEFLUOR® kit (StemCell Technologies Inc.) and is expressed as percentage of positive cells. B. Anchorage independent growth of PCSCs. Soft agar assays were carried out in 96 well plates using Cell Biolabs CytoSelect™ 96-well Cell Transformation Assay. 500, 2500 and 10000 cells were plated in triplicates. Upper panel shows 2 × lens low magnification pictures of the entire well (of a 96 well plate) at 2500 cells density after 10 days in culture. Lower plot presents the averaged relative fluorescent units for the three cell densities assessed at 10 days in culture. C. Kapplan-Meier survival curves of NOD/SCID mice xenograft experiments injecting parental PCSCs. 100, 1000, 10000 and 300000 cells of PCSC-1, PCSC-2 or PCSC-3 were used per injection as described in Materials and Methods. [file 1471-2164-11-324-S1.PDF]
