# Supplementary figures and images for: Genomic profiling of tumor initiating prostatospheres
Source: BMC Genomics. 2010 May 25;11:324. doi: 10.1186/1471-2164-11-324 (PMC2900264; doi:10.1186/1471-2164-11-324)

A. LNCaP Stem Cell Markers

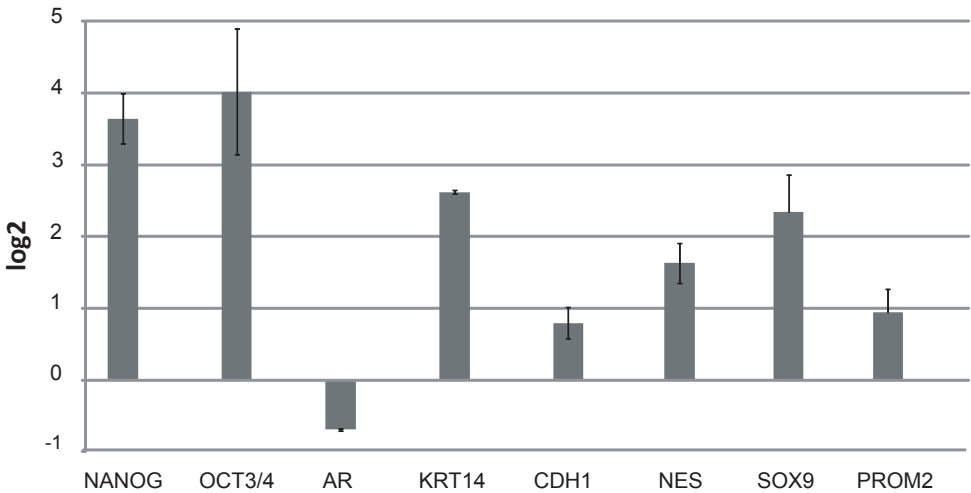

B. PCSCs Stem Cell Markers

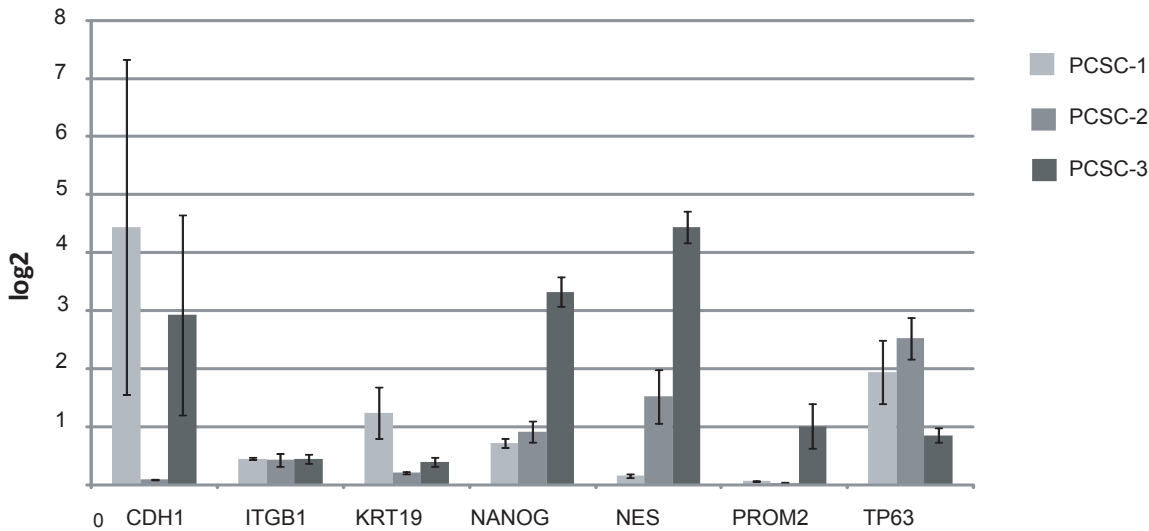

C. 66 gene signature

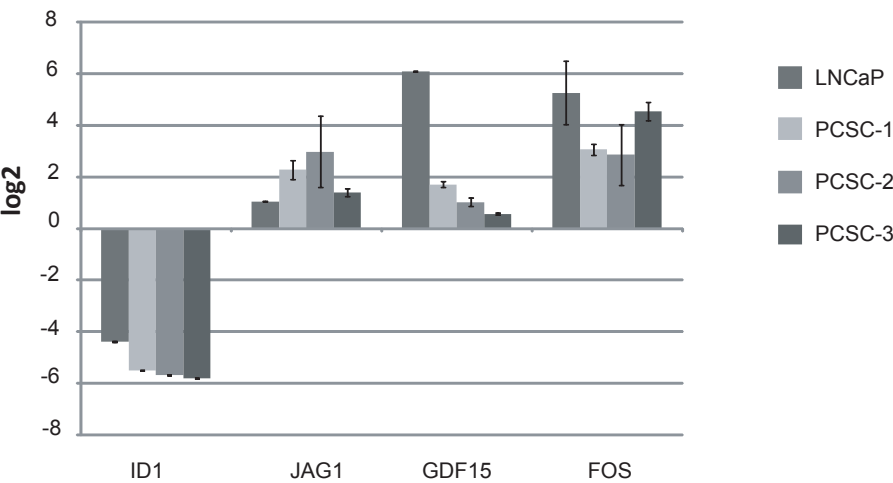

Supplement: Additional file 2 — qRT-PCR validation of selected genes. Stem cell markers in LNCaP (A) and PCSCs (B) and the 66 gene signature genes (C). The data for each gene is the average of at least two biological replicates. Error bars represent averaged standard error in the measurements of a given gene. [file 1471-2164-11-324-S2.PDF]

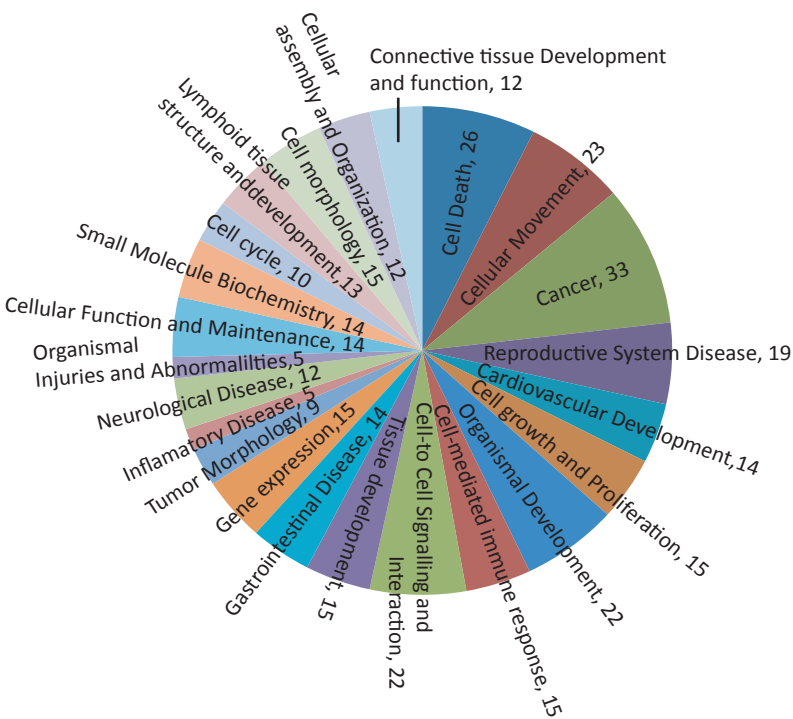

Supplement: Additional file 3 — Most significant (p ≤ E-4) functional categories represented in the 66 genes PS gene set. The numbers indicate the number of genes in each category. [file 1471-2164-11-324-S3.PDF]
