# Supplementary material for: Genomic profiling of tumor initiating prostatospheres
Source: BMC Genomics. 2010 May 25;11:324. doi: 10.1186/1471-2164-11-324 (PMC2900264; doi:10.1186/1471-2164-11-324)
Supplement: Additional file 4 — List of TF binding sites enrichments retrieved from MSigDB. Upper chart presents the five PSs that are common to LNCaP and PCSCs (≥2-fold change and p ≤ 0.01). Lower chart describes those TF that are specific for each type of cell line. [file 1471-2164-11-324-S4.PDF]

TRANSCRIPTION FACTOR OVERLAP LNCAP-PCSC

Description (Genes with promoter regions [-2kb,2kb] around transcription start site containing the motif indicated)	LNCaP # genes in overlap (k)	LNCaP k/K	LNCaP p value	PCSC # genes in overlap (k)	PCSC k/K	PCSC p value
CACGTG MYC: v-myc myelocytomatosis viral oncogene homolog (avian)	16	0.015	6.71E-02	22	0.0206	5.23E-03
CAGCTG REPIN1: replication initiator 1	17	0.0109	3.79E-03	36	0.0231	5.42E-03
CAGGTG TCF3: transcription factor 3 (E2A immunoglobulin enhancer binding factors E12/E47)	27	0.0105	1.24E-04	68	0.0264	9.38E-03
GCANCTGNY MYOD1: myogenic differentiation 1	11	0.0115	2.54E-02	20	0.021	9.12E-03
GCCATNTTG YY1: YY1 transcription factor	2	0.0045	0.00823	5	0.0113	0.0027

	LNCaP	PCSC
SPECIFIC UP	SP1,MAZ,FGI1,CART,ELK1,TCF1	IRF, NFAT,STAT5B, JUN,CREB1,DBP.SRF.POU1F1,TCF-1,HSF1,LEF1,TCF8
SPECIFIC DOWN	TGABPA,MAZ,LEF1,ESRRA,ELK1,PAX4,SOX5,JUN,POU2F1	SP1,ELK1, MAZ,TCF3,YY1,GABP,GATA1,ESRRA,SREBF-1,NF1,ATF3
